# Underestimated harmful effects of assays for detection of IgG antibodies against food

**DOI:** 10.1186/2045-7022-5-S3-P80

**Published:** 2015-03-30

**Authors:** Silar Mira, Zidarn Mihaela, Nina Celesnik Smodis, Peter Korosec

**Affiliations:** 1 The University Clinic of Respiratory and Allergic Diseases, Golnik, Slovenia

## Background

Measurement of food specific IgG antibodies by in vitro assays indicate a physiological response of the immune system after exposition to food. It is not related to food allergy or food intolerance. Unfortunately, many people do not know the difference. Due to the presence of IgG antibodies they decide for an unnecessary diet.

## Methods

We examined the Food Detective^TM^, quick blood test for determination of food IgG antibodies with a panel consisting of 46 food allergens, a positive control and a negative control. We tested 7 healthy controls and 14 patients (7 atopics and 7 with confirmed food allergy). All healthy controls had negative skin tests for wheat, milk, egg and meat.

## Results

All tested subjects were positive for at least 15% to maximum 48% of all tested allergens. We found 7 healthy controls positive for wheat and 4 out of 7positive for milk and egg. All of them were also positive for white fish mix (haddock, cod and plaice).

## Conclusions

In this study we showed that the major disadvantage of the test is its irrelevant results. Such tests should not be available in pharmacies.

